# Editorial: Endocrinological and Social Moderators of Emotional Well-Being During Perimenstrual, Perinatal and Perimenopausal Transitions: What Women Want for Sexual Health and Smooth Hormonal Changes

**DOI:** 10.3389/fpsyg.2021.813291

**Published:** 2022-02-02

**Authors:** Sophie Schweizer-Schubert

**Affiliations:** ^1^International Psychoendocrinology and Psychotherapy Practice, Heilbronn, Germany; ^2^Institute of Medical Psychology, University Hospital Heidelberg, Heidelberg, Germany

**Keywords:** psychoneuroendocrinology, reproductive mood disorders, depression, anxiety, stress, pregnancy, menopause, menstrual cycle

Navigating women's mental health between hormonal and social factors has only lately become a focus of research in our international psychoendocrinology community. Research had long been in men's hands; in female hands it started out stymied by contradicting forces—fear of losing the “gender race” in an honest view of biological differences between genders, most notably significant hormonal fluctuations during women's fertile years, vs. the urgent need to identify social(-interactional) parameters women require for well-being and fulfillment of potential, especially in times of hormonal transitions. Nowadays, gender problems for the average couple typically arrive with children and pressures to juggle motherhood and career. Unjust social-interactional patterns in gender roles could constitute a key factor for the significantly higher prevalence of depressive disorders in women vs. men. Stress resulting from social injustice has always compromised mental health. Women with a stress reactivity marked by trauma, either to themselves or (epi-)genetically transmitted, need our particular attention, and here first and foremost those women choosing motherhood next to a career yet lacking adequate support. Their vulnerability to falling into socially detrimental situations and further health problems may in turn lead them into a health service traditionally labeling them as psychiatrically ill or sexually dysfunctional when they are simply suffering from social-interactional injustice. Historically, mankind could have taken a fairer approach, addressing social-interactional stressors instead of additionally attacking female self-confidence by subjecting them to psychiatry. Lack of support to women has been identified as a highly relevant factor for women's higher prevalence of depression (Müters et al., 2013). Particularly a man's lack of emotional support to his female partner, including empathy (Kazmierczak et al., 2015) or humor as stress-relievers during hormonal transitions, increases female risk for depression. Also, a research agenda for women's mental health identified the following gaps in knowledge in a paper on the outcomes of the International Society of Psychosomatic Obstetrics and Gynecology (ISPOG) conference in The Hague: endometriosis, fertility management for cancer survivors, pregnancy deniers, incongruous childbirth expectations and experiences and care provision following birth trauma (Quinlivan et al., 2020).

Gynecological Psychology is still a relatively young field but has also grown in recent years with the addition of a psychoendocrinological focus. From a social-interactional perspective, women's adaptation to endocrine transition periods have been found related to male partners' hormonal fluctuations, a prominent example being the testosterone decline in men prior to childbirth reducing aggression and enabling their increased social support for their female partners such as sharing childcare and home duties (Saxbe et al., 2016). Findings suggest increased risk for female depression and anxiety when these co-fluctuations are out of sync in a couple.

With our article collection we hope to strengthen a Psychoendocrinological Approach to women's mental health that is historically rooted in the biopsychosocial model (Engel, 1977) as well as the stress vulnerability model (Zubin and Spring, 1977). We highlight hormonal fluctuations, biological vulnerability to stress and actual social stressors as key parameters in female depression, focusing on puberty, menstrual cycle, peripartum, perimenopause, alongside female sexual health aiding stress-release in women. We opted for three foci: the GABA-A receptor, central to stress mechanisms; fluctuating sex hormones and the neurosteroid Allopregnanolone; Oxytocin, a hormone at the heart of social interaction.

Alongside new data we provide a thorough review of the key genetic, neuroendocrinological and social-interactional perspectives on the connection between hormonal fluctuations, stress and mental ill-health. Schweizer-Schubert et al. summarize the current state of research on GABA-A receptor and Allopregnanolone, establishing common denominators of “Reproductive Mood Disorders” (depressive symptoms during the above-mentioned hormonal transitions). Which women experience increased sensitivity to fluctuations in sex steroids (estrogen, progesterone) and stress-related steroids? We integrate both dynamics into the concept of “steroid hormone sensitivity” highlighting the role of psychosocial stressors with the aim of providing a theoretical basis for more integrative endocrinological, social and psychological treatment options and prevention strategies for susceptible women.

From puberty and the start of the menstrual cycle, the prevalence of depression in women rises dramatically. McGuire et al. present data suggesting more advanced pubertal physical development and greater rejection sensitivity predicted higher levels of depressive symptoms later. Walsh et al. indicate the role of trauma finding that early life abuse increases effects of intranasal oxytocin on symptoms of premenstrual dysphoric disorder, highlighting the importance of examining women's social-interactional history for predicting hormonal responses. Leeners et al. investigate the association between estradiol, stress perception and stress-related cognitive performance during the menstrual cycle and fertility treatment. Poromaa et al.'s brain imaging study confirms low levels of Allopregnanolone relate to higher serotonergic binding in the prefrontal cortex, crucial to cognitive performance and top-down emotional regulation and the typical pre-ovulatory increase in female well-being.

Concerning peripartal mental health, Melón et al. provide further insights into the role of neurosteroids *via* preclinical testing of a novel therapeutic compound for postpartum depression. Osborne et al. focus on pregnancy's second trimester Allopregnanolone as predictor of post-partum anxiety, which may precede depression. Kress et al. provide a glimpse into their multi-method cohort study protocol (Dresden Study on Parenting, Work, and Mental Health), contributing to greater understanding of the role of social, work and stress factors in mental and somatic health and its long-term endocrinological and transgenerational correlates within the family.

As to women's perimenopausal mental health in their pilot study Gordon et al. provide support for the hypothesis that perimenopausal estradiol fluctuations increase women's sensitivity to psychosocial stress and vulnerability to depression. Mernone et al. examine sexual health in aging women, showing age and postmenopausal status to be negatively associated with sexual functioning, but also that psychosocial factors such as emotional support are crucial to female sexual well-being. In Dickenson et al. we learn about the role of mindfulness in sexual arousal. They examine a potential oxytocinergic neuroendocrine mechanism underlying the link between mindfulness and sexual arousal by looking at subjective and oxytocinergic responses to mindfulness and correspondingly to sexual arousal.

To conclude this collection of articles, a simple question and potential answers—could the removal of the remaining psychosocial stressors for those women still living in patriarchally tinted structures (once again exemplified by the Covid-19 pandemic driving mainly mothers out of the workforce) be the most obvious first step to reduce the significantly higher prevalence of female depression? Let us revisit depression and anxiety as identifiers for unmet human needs due to social ills. Key solutions could be significantly improved societal responses to the female hormonal transitions framing human reproduction and aging and a clear view on requirements for good female sexual health, a fundamental pillar of stress-release for both sexes. A worldwide behavioral change might be required toward a fair management of biological differences between men and women, the ignoring of which has come to deteriorate female well-being in matching fulfillment from motherhood and career. Instead of supporting pharmacological profits from anti-depressants, benzodiazepines and hormonal treatments we could opt for increasing societal profits by applying these latest psychoendocrinological findings to lift psychosocial interactions between the sexes to the next level of social justice. Translations of research into practice are well under way as evidenced by new psychoendocrinology practices, psychoendocrinology courses in medical and psychological faculties and increasing societal awareness of such scientific insights. More to come!

## Author Contributions

The author confirms being the sole contributor of this work and has approved it for publication.

## Conflict of Interest

The author declares that the research was conducted in the absence of any commercial or financial relationships that could be construed as a potential conflict of interest.

## Publisher's Note

All claims expressed in this article are solely those of the authors and do not necessarily represent those of their affiliated organizations, or those of the publisher, the editors and the reviewers. Any product that may be evaluated in this article, or claim that may be made by its manufacturer, is not guaranteed or endorsed by the publisher.
